# Effect of time and temperature on stability of progestagens, testosterone and cortisol in Asian elephant blood stored with and without anticoagulant

**DOI:** 10.1093/conphys/coz031

**Published:** 2019-06-24

**Authors:** Jaruwan Khonmee, Janine L Brown, Mu-Yao Li, Chaleamchat Somgird, Khajohnpat Boonprasert, Treepradab Norkaew, Veerasak Punyapornwithaya, Wei-Ming Lee, Chatchote Thitaram

**Affiliations:** 1 Department of Veterinary Bioscience and Veterinary Public Health, Faculty of Veterinary Medicine, Chiang Mai University, Canal Road, Chiang Mai, Thailand; 2 Center for Species Survival, Smithsonian Conservation Biology Institute, Remount Road, Front Royal, VA, USA; 3 College of Veterinary Medicine, National Chung-Hsing University, Xingda Road, Taichung, Taiwan, R.O.C; 4 Department of Companion Animal and Wildlife Clinic, Faculty of Veterinary Medicine, Chiang Mai University, Canal Road, Chiang Mai Thailand; 5 Center of Elephant and Wildlife Research, Chiang Mai University, Canal Road, Chiang Mai, Thailand; 6 Department of Food Animal Clinic, Faculty of Veterinary Medicine, Chiang Mai University, Canal Road, Chiang Mai, Thailand

**Keywords:** Asian elephant, cortisol, hormone degradation, progestagens, testosterone

## Abstract

The value of biological samples collected in the field is compromised if storage conditions result in analyte degradation, especially in warmer climates like Thailand. We evaluated the effects of time and temperature on immunoactive steroid hormone stability in Asian elephant (*Elephas maximus*) blood stored with and without an anti-coagulant before centrifugation. For each elephant (5 male, 5 female), whole blood was aliquoted (*n* = 2 ml each) into 13 red top (without anticoagulant) or purple top (with anticoagulant) tubes. One tube from each treatment was centrifuged immediately and the serum or plasma frozen at −20°C (Time 0, T0). The remaining 12 aliquots were divided into stored temperature groups: 4°C, room temperature (RT, ~22°C), and 37°C, and centrifuged after 6, 24, 48 and 62 h of storage. Serum and plasma concentrations of progestagens in females, testosterone in males and cortisol in both sexes were quantified by validated enzyme immunoassays. Steroid concentration differences from T0 were determined by a randomized complete block ANOVA and Dunnett’s tests. The only evidence of hormone degradation was cortisol and testosterone concentrations in serum stored at 37°C. Testosterone concentrations declined by 34% at 48 h and 52% at 62 h, cortisol was decreased by 19% after 48 h and 27% after 62 h at 37°C, respectively. None of the other aliquots displayed significant changes over time at any temperature. In conclusion, steroids appear to be stable in blood for nearly 3 days at room or refrigeration temperatures before centrifugation; steroids in samples with ethylenediaminetetraacetic acid were particularly stable. However, warmer temperatures may negatively affect steroids stored without anti-coagulant, perhaps due to red blood cell metabolism. Thus, under field conditions with no access to cold or freezer temperatures, collection of plasma is a better choice for elephants up to at least 62 h before centrifugation.

## Teaser

Hormonal results may be compromised in field samples maintained without cold storage. This study determined that progestagen, testosterone and cortisol concentrations in blood with anticoagulant were stable at 37°C up to 62 h. Without anticoagulant, progestagens also were stable, but cortisol and testosterone in blood decreased over time.

## Introduction

Asian elephants (*Elephas maximus*) are endangered throughout most of their natural ranges (EN-A2c, ver. 3.1; IUCN Red list 2009), with several populations heading toward extinction unless mitigating efforts are successful in stemming population declines. From studies on captive animals, much is known about elephant biology, particularly through analyses of serum or plasma hormones (Brown, 2014). Assessments of progestagens are key to monitoring female reproductive condition (Brown, 2014), whereas testosterone is useful in studying musth, a period characterized by temporal gland secretions, urine dribbling and more antagonistic behaviors (Rasmussen *et al*., 1984; Dickerman *et al*., 1997). Cortisol increases under acute and chronic stress conditions (Woolley *et al*., 2008; Ghosal *et al*., 2013; Boyle *et al*., 2015; Moltesen *et al*., 2016; Benítez-Dorta *et al*., 2017) and if prolonged, can suppress reproductive function (Wayland *et al*., 2002; Wingfield and Sapolsky, 2003; Breuner *et al*., 2008), leading to irregular cycling and acyclicity (Fanson *et al*., 2014). Cortisol increases during normal physiological states as well, including the follicular phase of the estrous cycle (Fanson *et al*., 2014) and musth (Brown *et al*., 2007). Non-invasive steroid monitoring methods (urine, feces, saliva, milk and hair) have been developed (Verkerk *et al*., 1998; Brown *et al*., 2010; Marcilla *et al*., 2012; Mack and Fokidis, 2017; Pawluski *et al*., 2017; Rakotoniaina *et al*., 2017), with feces being particularly well suited for field studies. However, under some circumstances (e.g. collections under field anesthesia), measures of circulating hormones in serum or plasma are desired.

Stability of hormones in blood varies among species, sample types and preservation methods (Wiseman *et al*., 1983; Abal *et al*., 1996; Taylor and Schuett, 2004; Hegstad-Davies, 2006; Jones *et al*., 2007; Tahir *et al*., 2013). It is recommended that blood be centrifuged soon after collection to obtain serum or plasma, and frozen immediately. However, for samples collected in the field, it can take hours or even days to reach laboratory processing facilities. Little is known about how steroids degrade in elephant blood, so the goal of this study was to determine the effects of storage time and temperature on immunoactive stability of steroids in Asian elephant blood: progestagens in females, testosterone in males and cortisol in both sexes.

## Materials and methods

### Animals and sample collection

This study was approved by the Faculty of Veterinary Medicine, Chiang Mai University, Animal Care and Use Committee (FVM-ACUC; permit number S39/2559). Female (*n* = 5; age range, 9–35 yr; mean, 19.6 ± 10.6 yr) and male (*n* = 5; age range, 15–50 yr; mean, 26.2 ± 14.0 yr) Asian elephants were housed at the Baan Chang Elephant Camp in northern Thailand (latitude, 19°06′51.6“N; longitude 98°53’39.2”E). Elephants were fed primarily corn stalk*, napier grass* (*Pennisetum purpureum*) and bana grass (*P. purpureum* X, *Petalophyllum americanum* hybrid) with regular access to water. Elephants participated in tourist activities, including bareback riding, bathing and feeding, and were in good health at the time of the study based on physical examinations by elephant camp veterinarians. Blood samples were collected from an ear vein by elephant camp staff or Chiang Mai University veterinarians.

In Study 1, 30 ml of blood was collected from each elephant using a 22-gauge IV catheter and 50-ml syringe between 1000–1100 h. Blood in 2-ml aliquots was divided among 13 red top tubes without anticoagulant (serum) and was kept in a styrofoam box with ice (maintained ~4°C) for transportation to Chiang Mai University. Upon arrival at the laboratory (<4 h), one tube from each elephant was centrifuged at 1500× g for 10 minutes, representing Time 0 (T0). The other 12 tubes were centrifuged after 6, 24, 48 and 62 h of storage at 4°C, room temperature (~22°C), or in a 37°C controlled temperature chamber (typical ambient temperature in the warm season).

**Table 1:** Mean ± SD and range values for progestagens, testosterone and cortisol concentration in samples at T0 in female (*n* = 5) and male (*n* = 5) Asian elephants in Thailand

**Hormone**	**Number of elephants**	**Serum**	**Plasma**
Progestagens (ng/ml)	5	3.16 ± 2.20 (0.26–5.99)	2.80 ± 1.79 (0.34–5.00)
Testosterone (ng/ml)	5	1.85 ± 1.19 (0.58–3.3)	1.88 ± 1.48 (0.35–3.97)
Cortisol (ng/ml)			
Male	5	1.12 ± 0.50 (0.65–1.73)	0.97 ± 0.61 (0.33–1.88)
Female	5	0.60 ± 0.28 (0.26–1.00)	0.56 ± 0.35 (0.22–1.12)

Study 2 was conducted 2 days later using the same elephants, with 30-ml of blood aliquoted into 13 purple top tubes with ethylenediaminetetraacetic acid (EDTA) anticoagulant (plasma), and the plasma harvested after 0, 6, 24, 48 and 62 h of storage at 4°C, RT or 37°C before centrifugation.

Serum and plasma samples (0.5–0.8 ml) were stored at −20°C until hormone analyses.

### Hormone analysis

All chemicals were obtained from Sigma Chemical Company (St. Louis, MO), unless otherwise stated. Concentrations of testosterone and cortisol in males, progestogens and cortisol in females were quantified by enzyme immunoassays (EIAs) validated for elephants using antibodies for progesterone (monoclonal, CL425; Brown *et al*., 2004), testosterone (polyclonal, R156/7; Somgird *et al*., 2016) and cortisol (polyclonal, R4866; Somgird *et al*., 2016). The monoclonal progesterone antibody crossreacts with reduced pregnanes present in elephant serum (Brown, 2014), and are herein referred to as ‘progestagens’. Briefly, 96-well plates (catalog no. 07–200-39; Fisher Scientific, Pittsburgh, PA, USA) were pre-coated with secondary antibody diluted in coating buffer (catalog no. X108, 20X; Arbor Assays, Ann Arbor, MI): 150 μl (10 μg/ml) goat anti-mouse IgG for progesterone, and goat anti-rabbit IgG (Arbor Assays) for cortisol and testosterone EIAs. Coated plates were prepared by incubating at RT for 15–24 h. Wells were emptied and blotted dry, followed by adding 250 μl blocking buffer (100 mM phosphate, 150 mM sodium chloride, 1% Tween20, 0.09% sodium azide, 10% sucrose, pH 7.5) and incubating for 15–24 h at RT. After incubation, wells were emptied, blotted and dried in a Sanpla Dry Keeper (Sanplatec Corp., Auto A-3, Japan) with loose desiccant in the bottom. After drying (humidity <20%), plates were heat-sealed in a foil bag with a 1-g desiccant packet, and stored at 4°C until use.

Samples or standards (50 μl) (progestagens: Sigma Diagnostics Cat. #P0130, range 0.78–200 pg/well; testosterone: Steraloids Cat. #A6950, range 2.3–600 pg/well; cortisol: Sigma Diagnostics Cat. #H4001, range 3.9–1000 pg/well) were added to appropriate wells. Next, 25 μl of steroid horseradish peroxidase conjugate (HRP; progestagens 1:40000 dilution; testosterone 1:10000 dilution; cortisol 1:16000 dilution) was immediately added to each well, followed by 25 μl of primary antibody (progestagens, 1:10000 dilution; testosterone, 1:8500 dilution; cortisol, 1:75000 dilution) added to all but non-specific binding wells and incubated at RT for 1 h. Plates were then washed four times with wash buffer (1:20 dilution, 20X Wash Buffer Part No. X007; Arbor Assays, MI) and 100 μl of TMB substrate solution was added, followed by incubation for 45–60 min at RT without shaking. The absorbance was measured at 405 nm by a microplate reader (TECAN). Assay sensitivities were 0.078, 0.047 and 0.078 ng/ml for progestagens, testosterone and cortisol, respectively. Samples were analyzed in duplicate; inter- and intra-assay coefficients of variation were <10% and <15% (*n* = 6 for progestagens, *n* = 5 for testosterone, *n* = 11 assays for cortisol), respectively. Progesterone, testosterone and cortisol EIAs were validated in Thailand for elephant serum by demonstrating parallelism between serial dilutions of neat serum or plasma and the respective standard curves (Pearson’s correlation coefficients, *r* > 0.95).

**Figure 1:**
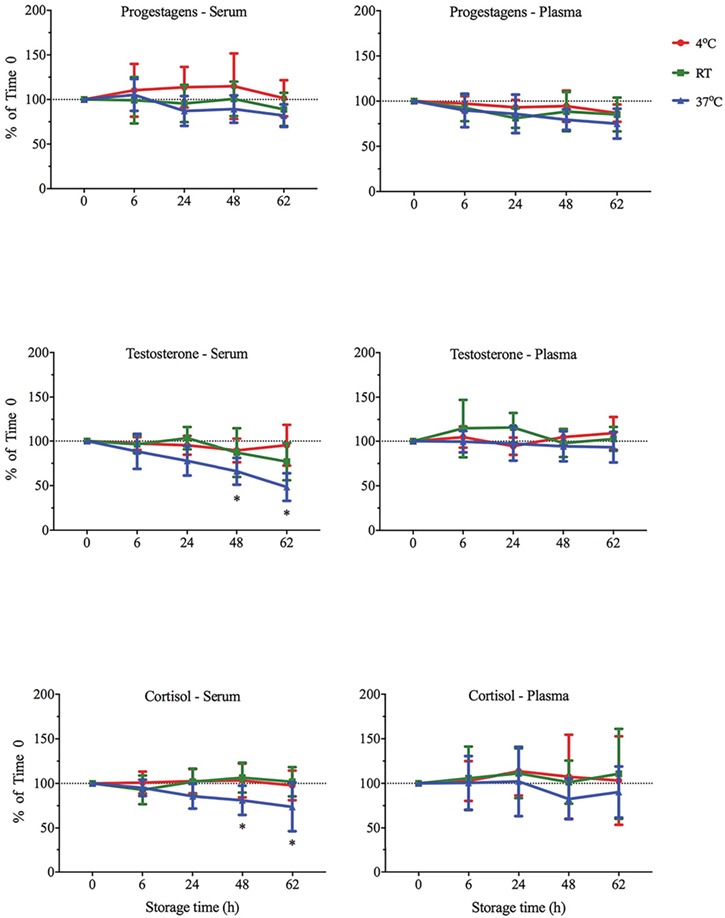
Mean ± SD concentrations of progestagen (*n* = 5), testosterone (*n* = 5) and cortisol (*n* = 10) in elephant serum and plasma samples stored at 4°C, RT (~22°C) and 37°C for up to 62 h before centrifugation Data are expressed as a percentage of T0 values. For each treatment, asterisks denote values that differ from the initial T0 concentration (*P* < 0.05).

### Statistical analyses

Aliquots from the same animals were assigned as a block following a randomized complete block design. Each aliquot was randomly assigned to a time and temperature treatment. Hormone concentrations were converted into percentages of T0 values by the following equation: [concentration Tx (*x* = 6, 24, 48, 62)]/(concentration T0). Percentage data (*n* = 5 for testosterone and progesterone, *n* = 10 for cortisol; no missing data points) are presented as mean ± standard deviation (SD). The effect of time (0, 6, 24, 48 or 62 h) on hormone concentration was assessed using a randomized complete block ANOVA, with concentration as the dependent variable and time as a fixed effect. Separate models were run for each substrate, temperature and hormone combination. Normality of residuals was evaluated by plotting QQ graphs, and the homogeneity of variance assessed by plotting residuals and fitted values. Most models did not violate normality and homogeneity of variance assumptions; however, slight deviations from a normal distribution were observed in some models as evidenced by residuals deviating from a straight line. Results were still used because ANOVA is particularly robust to normality problems (Glass et al. 1972; Harwell et al. 1992: Lix et al. 1996; Khan and Rayner, 2003; Blanca *et al.,* 2017). If time was significant at *P* < 0.05, a *post hoc* Dunnett’s test was used to compare differences in hormone concentrations between time points. Statistical significance was set as α = 0.05. All statistical analyses were performed using R version 3.4.4 (R Development Core Team, 2015).

## Results

### Hormone concentrations

Descriptive data are presented in Table 1, highlighting the variability in mean and mean range progestagens, testosterone and cortisol values across individuals. Progestagen concentrations were above baseline (0.05 ng/ml) indicating females were in the luteal phase of the cycle (Brown *et al.,* 2004). Bulls were not in musth, as reflected by testosterone values <5 ng/ml (Brown *et al.,* 2007).

### Study 1

Progestagen concentrations in female serum were not significantly affected by either storage time or temperature.

Testosterone concentrations in male serum did not change significantly when stored at 4°C or RT. However, time did have a significant effect on testosterone concentrations when stored at 37°C (F_4,16_ = 11.99, *P* = 0.0001). *Post-hoc* comparisons indicated that testosterone concentrations were 34% lower than T0 after 48 h (t_16_ = 4.16, *P* = 0.0027), and 52% lower after 62 h (t_16_ = 6.33, *P* = 0.0001; Fig. 1).

In addition, cortisol concentrations in male and female serum did not change significantly when stored at 4°C or RT; however, time did have a significant effect on cortisol concentrations when stored at 37°C (F_4,36_ = 4.43, *P* = 0.0051). *Post-hoc* comparisons indicated that cortisol concentrations were 19% lower than T0 after 48 h (t_36_ = 2.996, *P* = 0.017), and 27% lower after 62 h (t_36_ = 3.624, *P* = 0.0003; Fig. 1).

### Study 2

There were no significant time and temperature of storage effects on concentrations of plasma steroids relative to T0 (Fig. 1).

## Discussion

This study investigated the impact of temperature and time on steroid hormone (progestagens, testosterone and cortisol) degradation in blood of male and female Asian elephants stored with or without anticoagulant before centrifugation, and found that storage at 4°C or RT (~22°C) for at least 62 h had little impact on serum or plasma concentrations. All steroids in blood with anticoagulant were not significantly different from T0 when stored at 37°C for up to 62 h before plasma harvesting. By contrast, both testosterone and cortisol in serum stored at 37°C declined significantly within 48 h, ~34% for testosterone and 20% for cortisol, and by 62 h levels were only half to a quarter of original levels, respectively. These findings agree with reports in some species, but not others, and highlight species and steroid differences in hormone stability between sample types.

Studies on the stability of progesterone in blood have yielded mixed results. While some found progesterone to be quite stable, human serum unaltered after 48 h at 22°C (Wiseman *et al*., 1983; Diver *et al*., 1994; Jones *et al*., 2007) and dog serum and plasma (EDTA) stable for 2 weeks at 20–22°C (Tahir *et al*., 2013), others found that progesterone degrades rapidly. For example, progesterone in dog serum (2 h) declined more quickly than in dog plasma (heparin; 5 h) at 4°C (Volkmann, 2006). In cows, serum or plasma (heparin) progesterone decreased 50% within 24 h at 22°C (Wiseman et al., 1983) and > 70% after 72 h at 22–26°C (Reimers *et al*., 1983). In another study, serum progesterone was only 40% of initial concentrations after 8 h and < 10% after 24 h at varying temperatures (De Castro *et al*., 2004). Declining progesterone concentrations in blood before centrifugation may be due to the presence of blood cells and effects on steroid metabolism (Ohtsuka and Koide, 1969; Vahdat *et al*., 1981, 1984). Cytochrome P-450 in lymphocytes and platelets also can metabolize steroids (Lemberg and Barrett 1973; Hodgson and Guthrie, 1980). However, in elephants, degradation of progestagens in serum or plasma was not observed at any storage temperature, at least up to 62 h, suggesting blood cell steroid metabolism of this steroid did not occur during that time. Perhaps this is related to 5α-reduced pregnanes (e.g. 5α-pregnane-3,20-dione, 5α-pregnane-3-ol-20 one, 17α-hydroxyprogesterone) being the predominant luteal steroids, rather than progesterone (Hodges, 1998).

Testosterone concentrations in elephant bull samples stored at 4°C and RT were stable in serum and plasma for at least 62 h, but decreased within 48 h in samples stored at 37°C without anticoagulant. In goats, testosterone was stable in samples with fluoride-potassium oxalate for at least 24 h at 22°C (Fahmi *et al*., 1985). Similarly, in diamondback rattlesnakes, testosterone concentrations in plasma were unchanged during storage at 0°C for up to 24 h (Taylor and Schuett, 2004). However, concentrations in that study were equally stable at 40°C, and did not show the decline observed in elephants at an elevated temperature (37°C in our study). Testosterone in human blood exhibited no clinically relevant changes during storage at RT for 168 h (Diver *et al*., 1994). However, a more recent study found testosterone concentrations in human samples without anticoagulant actually increased within the first 48 h of storage at 22°C (Jones *et al*., 2007). Similarly, in dogs, testosterone in plasma stored at RT were unchanged for up to 144 h, but in serum, concentrations were increased at 48 h (Frankland, 1985). Thus, there can be differences in steroid immunoactive stability between samples stored with and without anticoagulant, with serum values being influenced more.

Cortisol concentrations in the blood of cows with and without anticoagulant were stable at 25°C for at least 62 h (Reimers *et al*., 1983), and at 4°C for up to 40 h in the blood of dogs with (EDTA, heparin) or without anticoagulant (Olson *et al*., 1981), and at RT in gray seals (Bennett *et al*., 2012), consistent with the results of this study. By contrast, cortisol in human blood stored in heparin was increased by ~15% in plasma samples stored at RT by 48 h, and at 4°C by 148 h (Diver *et al*., 1994).

## Conclusion

Immunoactive concentrations of progestagens, testosterone and cortisol in blood stored with anticoagulant were not significantly different from T0 over time, and exhibited no significant changes when stored at 4°C, RT or 37°C for up to 62 h. For blood without anticoagulant, serum progestagens also were not significantly different from T0 across all temperatures and times of storage. However, serum cortisol and testosterone showed significant decreases in concentrations at 48 and 62 h of storage at 37°C.
